# Ecological composite fertilizer application enhances wheat yield and optimizes rhizosphere microbial community under reduced fertilization

**DOI:** 10.3389/fpls.2026.1771450

**Published:** 2026-02-02

**Authors:** Yu Wang, Xinhao Luo, Meiling Ping, Haining Wang, Yueming Bao, Chuansheng Zhao, Xiaoyu Li, Jin Chen

**Affiliations:** 1 Schools of Life Sciences, Anhui Agricultural University, Hefei, China; 2 National Engineering Laboratory of Crop Stress Resistance Breeding, Anhui Agricultural University, Hefei, China

**Keywords:** Ecological composite fertilizer, fertilizer reduction, high-throughput sequencing, Rhizosphere microbial community, wheat yield

## Abstract

Excessive fertilization poses a major threat to sustainable agriculture, resulting in resource waste and environmental degradation. The ecological composite fertilizer (ECF) combined with fertilizer reduction represents a promising strategy to improve rhizosphere microbial diversity in wheat systems. A field experiment, containing six treatments, namely traditional compound fertilizer (TF, applied at the conventional rate) with a 10% reduction (TF90), TF90 plus ECF application (TF90+ECF), TF with a 15% reduction (TF85), TF85 plus ECF application (TF85+ECF), TF with a 20% reduction (TF80), and TF80 plus ECF application (TF80+ECF), was conducted to explore the influences of fertilizer reduction combined with ECF application on wheat yield and rhizosphere soil microbial diversity. Results showed that the TF85+ECF treatment achieved the highest wheat yield at 8,717.33 kg ha^−1^, which was significantly greater than all other treatments and represented a 30.63% increase over the TF85 treatment. The TF85+ECF group significantly enhanced the activities of the carbon and nitrogen cycling enzymes β-1, 4-glucosidase glucosidase (BG) and urease (UE), and increased the abundances of the functional genes *cbbLR* and *amoA*. In the +ECF treatment groups (TF90+ECF, TF85+ECF, and TF80+ECF), linear discriminant analysis effect size (LEfSe) and specialization-occupancy (SPEC-OCCU) analyses identified keystone microbial taxa, including positively correlated taxa with biocontrol and metabolic versatility (e.g., *Trichoderma, Solicoccozyma*) and negatively correlated potential pathogens (e.g., *Alternaria*). Co-occurrence network analysis revealed that the TF85+ECF group streamlined bacterial network architecture while enhanced fungal network complexity and connectivity. Mantel tests and correlation analyses indicated that soil organic carbon, BG activity, and *cbbLR* gene abundance were significantly linked to microbial community structure, and keystone taxa were strongly correlated with soil nutrient cycling functions. Our findings provide a microbiome-based strategy and a novel perspective for sustainable wheat production and targeted microbial management in agriculture.

## 1 Introduction

Soil serves as a critical “seed bank” for plant-associated microbiomes, fundamentally governing the assembly and functional dynamics of microbial communities associated with fungal hyphae and plant roots (Goldmann et al., 2020; Yuan et al., 2021). In terrestrial ecosystems, soil microorganisms play vital roles in regulating energy flow, nutrient cycling, and sustaining soil productivity processes that are essential for crop yield and ecosystem stability (Ye et al., 2021). Changes in microbial community structure and diversity are widely regarded as sensitive indicators of soil health, and their response to agricultural management provides key insights into soil functional integrity (Larkin, 2003). A prominent example is the Shajiang black soil of the Huaihe River Basin, a major grain-producing region where long-term intensive cultivation has led to progressive soil degradation (Wang et al., 2021; Wang and Wang, 2022). Therefore, developing integrated agricultural strategies that enhance crop productivity while promoting environmental sustainability in this region is urgently needed.

Wheat (*Triticum aestivum* L), the most extensively cultivated cereal crop worldwide, supplies approximately 20% of global dietary protein and energy, rendering it indispensable for global food security (Bailey Serres et al., 2019; He et al., 2025). However, contemporary wheat production confronts substantial sustainability challenges, particularly stemming from soil degradation and ecological deterioration caused by excessive chemical fertilizer application (Zhang et al., 2015; Liu et al., 2020). Since the 1960s, global nitrogen fertilizer utilization has increased nearly tenfold, imposing unprecedented stress on agricultural ecosystems (Liu et al., 2010). The ramifications of excessive fertilization manifest through multiple pathways: fundamental alterations in soil bacterial and fungal community architecture, dramatic reductions in beneficial microbial populations, suppression of soil enzyme activities, and disruption of essential nutrient cycling processes (Xu et al., 2012). These transformations critically undermine agricultural sustainability by threatening yield stability and elevating food safety concerns (Tilman et al., 2002). At present, the comprehensive elucidation of the mechanisms and integrated effects of green fertilization strategies on the synergistic enhancement of soil health and crop productivity remains incomplete.

The adoption of environmentally sustainable practices to improve soil health represents a critical strategy for enhancing soil nutrients, fostering a healthy farmland environment, and safeguarding global food security (Efthimiou, 2025). In this context, the ecological composite fertilizer (ECF), which integrates biochar (BC) with arbuscular mycorrhizal fungi (AMF), has emerged as a promising approach to synergistically improve rhizosphere properties and plant performance (Li et al., 2025). The porous structure and high adsorption capacity of BC enable the amendment of soil physical properties and enhancement of nutrient retention (Tian et al., 2025). AMF form symbiotic associations with host plants, extending root nutrient acquisition and contributing to soil aggregate stability through their extensive hyphal networks (Kuila and Ghosh, 2022; Sun and Shahrajabian, 2023). For example, in heavy metal-contaminated soils, BC and AMF co-application significantly modifies rhizosphere microbial community structure, enhances its diversity and stability, and regulates plant metal uptake and translocation (Li et al., 2025). Similarly, in soils with continuous cropping obstacles, such combinations enrich beneficial microbial networks centered on AMF (e.g., *Rhizophagus*) and biocontrol agents (e.g., *Trichoderma*, *Bacillus*), suppressing pathogens by upregulating beneficial rhizosphere amino acid metabolism (Tian et al., 2025). The synergistic interaction between BC and AMF has been shown to enhance plant physiological performance and stress tolerance, contributing to improved crop outcomes (Augé, 2001). However, most existing research has focused on the effects of ECF under conventional fertilization regimes or specific abiotic stressors. Consequently, the systematic influence and underlying mechanisms of ECF under graded reductions in fertilizer application, which is a core strategy for sustainable agricultural intensification, remain poorly understood. Specifically, it is still unclear how ECF interacts with fertilizer reduction to reconfigure the diversity, network topology, and functional gene expression of the rhizosphere microbiome in staple crops such as wheat.

In this study, a field experiment was conducted to investigate the effects of ECF application combined with gradient fertilizer reduction on wheat yield and the rhizosphere soil microbial communities, with a focus on their diversity, composition, co-occurrence networks, and environmental coupling. Specifically, the aims were to: (1) elucidate the impacts of ECF under fertilizer reduction on wheat growth and the structural and functional responses of the rhizosphere microbiome; (2) decipher the relationship between the topological features of rhizosphere microbial co-occurrence networks and soil nutrient cycling functions, and to identify the keystone microbial taxa involved. This study provides novel insights into optimizing fertilization strategies and enhancing agroecosystem sustainability in wheat production systems of the HuangHuaiHai Plain and analogous regions.

## 2 Materials and methods

### 2.1 Experimental design

A field experiment was conducted at the Agricultural Technology Demonstration Farm in Mengcheng County, Anhui Province, China (33°16′N, 116°55′E) (**Supplementary Figure S1a**), a region characterized by a warm temperate, semi-humid monsoon climate with a mean annual temperature of 14.8°C and an average annual precipitation of approximately 732.63 mm, concentrated primarily from July to September. The cropping system follows a winter wheat-summer maize rotation, achieving two harvests per year. The soil was classified as Shajiang black soil, a calcareous alluvial lacustrine sedimentary soil, and corresponds to Calcaric Vertisols (WRB) or Typic Calciusterts (USDA Soil Taxonomy). The topsoil (0–20 cm) had the following initial physicochemical properties: pH 7.24, bulk density 1.35 g cm^−3^, organic carbon 11.5 g kg^−1^, total nitrogen 1.29 g kg^−1^, total phosphorus 0.39 g kg^−1^, total potassium 7.91 g kg^−1^, alkali-hydrolyzable nitrogen 146 mg kg^−1^, available phosphorus 18.9 mg kg^−1^, and available potassium 162 mg kg^−1^.

The experiment was conducted in 2024 with three repetitions per treatment (2 m × 2 m per plot) under a randomized block design. The conventional fertilization rate of traditional compound fertilizer (TF) was 225 kg N ha^−1^, 112.5 kg P_2_O_5_ ha^−1^, and 112.5 kg K_2_O ha^−1^. This study comprised six treatments: TF with a 10% reduction (TF90), TF90 plus ECF application (TF90+ECF), TF with a 15% reduction (TF85), TF85 plus ECF application (TF85+ECF), TF with a 20% reduction (TF80), and TF80 plus ECF application (TF80+ECF). These treatments were based on three levels of total NPK reduction relative to the conventional TF rate. The ECF used in this study, defined by the co-application of biochar and AMF, was applied at a rate of 0.035 g biochar and approximately 10 viable AMF spores per plant, a dosage selected based on effective root colonization and plant growth promotion observed in preliminary pot trials (Li et al., 2025). The biochar was produced by pyrolyzing wheat straw at 500°C for 2 h and had the following properties: pH 9.46 (1:10 w/v), total C 42.21%, total N 8.34%, total P 2.31%, total K 16.12%, ash content 7.23%, cation exchange capacity 19.4 cmol kg^−1^, and specific surface area 246.18 m² g^−1^. The AMF strains (*Rhizophagus irregularis*, BGC DAOM 197198) were provided by Anhui Agricultural University. The ECF was applied once at sowing via in-furrow placement (3–5 cm depth) alongside the seeds. Wheat cultivar “Wofengmai 169” was selected for this study, with a planting density of 2.25 million plants ha^−1^. For each treatment, half of the compound fertilizer was applied as a basal dose before sowing, and the remaining half was top-dressed at the jointing stage. All other field management practices (irrigation, weeding, and pest control) were uniformly applied across all plots according to local conventional methods.

### 2.2 Sample collection and processing

Plant samples were collected at three developmental periods: jointing, flowering, and maturity. At each period, five uniformly growing plants were randomly selected per plot, yielding a total of 15 plants per treatment. Agronomic traits-including plant height, stem diameter, root dry/fresh weight ratio, and spike number were measured on these samples. At maturity period, concurrent sampling of rhizosphere soil and roots was performed (**Supplementary Figure S1b**). Soil was collected from 0–20 cm depth within 2 mm of main roots using sterile shovels, with five composite samples obtained per plot. Roots were gently rinsed with deionized water, segmented, and fixed in FAA solution (formalin-acetic acid-alcohol, 5:5:90, v/v/v) at 4°C for ≤48 h for subsequent assessment of AMF colonization rates (**Supplementary Figure S1c**). Each composite soil sample was divided into three aliquots for distinct analyses: (1) air-dried at 25°C for physicochemical characterization, (2) stored at 4°C for microscopic quantification of AMF spore density, and (3) lyophilized and stored at -80°C for microbial DNA extraction and high-throughput sequencing (**Supplementary Figures S1d–f**).

### 2.3 Agronomic trait quantification and AMF colonization assessment

Wheat phenotypes were quantified at distinct developmental stages. Plant height was measured using a graduated ruler, and stem diameter was determined at the third internode with vernier calipers. Shoot samples were separated and weighed to obtain fresh weight (FW), then oven-dried at 105°C for 30 minutes followed by drying at 70°C to constant weight for dry weight (DW) determination. The root dry/fresh weight ratio was calculated as DW/FW. Grain yield assessment was conducted at maturity period by harvesting three independent 1 m² areas per treatment as biological replicates. Total ears per plot were counted, and grain yield per square meter was determined. Thirty representative ears per treatment were randomly selected for measuring grains per ear (GPE) and thousand grain weight (TGW). Soil AMF spore density was quantified through wet sieving followed by microscopic enumeration (Boyno et al., 2023). For root colonization assessment, FAA-fixed root segments underwent sequential processing: clearing in 10% KOH at 90°C for 1 h, acidification in 5% lactic acid with 5 min shaking, staining with 0.05% trypan blue for 24 h, and destaining in lactic acid-glycerol solution until tissue discoloration. Ten randomly selected root segments per slide (five slides per treatment) were mounted for microscopic examination. AMF colonization frequency (F%) was calculated as: F% = (colonized segments/total segments) × 100% (Huang et al., 2024).

### 2.4 Soil physiochemical analysis

Soil organic carbon (SOC) was quantified using the potassium dichromate oxidation method, total nitrogen (TN) was determined by Kjeldahl digestion, and available phosphorus (AAP) was measured via antimony molybdenum blue colorimetry. The activities of carbon-cycling enzyme β-1,4-glucosidase (BG), nitrogen-cycling enzyme urease (UE), and phosphorus-cycling enzyme alkaline phosphatase (ALP) were assessed using commercial spectrophotometric microplate assay kits (Suzhou Keming, China). Absorbance values were recorded at 400 nm for BG, 630 nm for UE, and 570 nm for ALP using a microplate reader (PerkinElmer, USA). Soil pH was measured potentiometrically with a digital pH meter (PB-10, Sartorius, Germany) in a 2.5:1 (w/v) water to soil suspension.

### 2.5 DNA extraction and high-throughput sequencing

Total microbial genomic DNA was extracted from 0.5 g soil samples using the PowerSoil^®^ DNA Isolation Kit (MoBio Laboratories, USA). DNA concentration and purity were assessed using a Nanodrop 2000 spectrophotometer (Thermo Fisher, USA; acceptable thresholds: A260/A280 = 1.8-2.0, A260/A230 > 1.7) and Qubit 3.0 Fluorometer (Invitrogen, USA). The V3-V4 hypervariable regions of the bacterial 16S rRNA gene were amplified with primers 338F (5′-ACTCCTACGGGAGGCAGCAG-3′) and 806R (5′-GGACTACHVGGGTWTCTAAT-3′), while the fungal internal transcribed spacer (ITS) regions were amplified using primers ITS1F (5′-CTTGGTCATTTAGAGGAAGTAA-3′) and ITS2R (5′-GCTGCGTTCTTCATCGATGC-3′). Amplification was performed in 25 μL reactions containing 12.5 μL of Phusion^®^ High-Fidelity PCR Master Mix (New England Biolabs), 2 μL of template DNA, 1 μL each of forward and reverse primers (10 μM), and nuclease-free water to 25 μL. The thermal cycling protocol comprised an initial denaturation at 95°C for 5 min; 35 cycles of 95°C for 30 s, 55°C for 30 s, and 72°C for 30 s; and a final extension at 72°C for 10 min. Purified amplicons were subjected to paired-end sequencing on an Illumina MiSeq platform (Major Bio-Pharm, China). Raw sequencing reads were processed through the following bioinformatic pipeline: quality filtering was performed with Fastp (v0.23.2), paired-end reads were merged using FLASH (v1.2.11; minimum overlap 10 bp, maximum mismatch rate 0.2%), and subsequent denoising, chimera removal, and amplicon sequence variant (ASV) calling were conducted with DADA2 (v1.18). Taxonomic classification was assigned using the classify-sklearn classifier against the SILVA 138 (for 16S) and UNITE v8.0 (for ITS) reference databases, with removal of mitochondrial and chloroplast sequences. All samples were rarefied to 10,000 sequences per sample for subsequent alpha and beta diversity analyses based on ASVs. The copy numbers of bacterial 16S rRNA genes, fungal ITS regions, and key functional genes for carbon (*cbbL*), nitrogen (*amoA*), and phosphorus (*phoD*) cycling were quantified via quantitative PCR (qPCR) across all soil treatments, as detailed in **Supplementary Table S1**.

### 2.6 Identification of keystone taxa

Microbial taxa exhibiting significant differences across distinct habitats were identified using the Linear Discriminant Analysis Effect Size (LEfSe) method. The discriminative power of each taxon was assessed through Linear Discriminant Analysis (LDA), with an LDA score threshold of > 3.0 set for identifying potential microbial biomarkers. Potential keystone taxonomic units within the community were evaluated using the SPEC-OCCU (Specific-Occurrence) method, which calculates taxon specificity and occupancy based on their distribution across environments and habitat specificity. From the top 1000 most abundant amplicon sequence variants (ASVs), those exhibiting both specificity and occupancy values ≥ 0.75 were classified as habitat specific taxa. Microbial co-occurrence networks were further analyzed to identify core microbial taxa based on within module connectivity (Zi) and among module connectivity (Pi). Taxa were categorized into three topological roles: module hubs (Zi > 2.5 and Pi ≤ 0.62), network hubs (Zi > 2.5 and Pi > 0.62), and connectors (Zi ≤ 2.5 and Pi > 0.62) (Chen et al., 2019).

### 2.7 Statistical analysis

Preliminary data organization was performed using Microsoft Excel. Agronomic and physiological parameters were subjected to two-way analysis of variance (ANOVA) with least significant difference (LSD) *post-hoc* tests in GraphPad Prism v9.0 (GraphPad Software, USA), with statistical significance defined at *P* < 0.05. Microbial alpha diversity indices (Chao1, Shannon, and Pielou) were calculated at the genus level using the Majorbio Cloud Platform (www.majorbio.com). Beta diversity patterns within bacterial and fungal communities were assessed based on Bray-Curtis dissimilarity matrices. A phylogenetic tree was constructed using the top 100 core microbial species ranked by relative abundance, and visualized and annotated through the Interactive Tree of Life (iToL, http://itol.embl.de) platform. Microbial co-occurrence networks were constructed and analyzed using the Integrated Network Analysis Pipeline (iNAP, https://inap.denglab.org.cn), with subsequent network visualization performed in Gephi v0.10.1. Key topological properties-including total nodes, total edges, average degree, average clustering coefficient, and modularity index were computed using built in algorithms (Yang et al., 2023). All statistical analyses and visualizations were implemented in R (version 4.2.2). To elucidate relationships between microbial communities and ecosystem functioning, Spearman’s rank correlation analysis and random forest modeling (using the “random Forest” package) were applied, with significance thresholds set at |r| > 0.95 and *P* < 0.01 (Yang et al., 2022). Mantel tests (“vegan” package) were further employed to evaluate correlations between microbial community composition and key environmental factors.

## 3 Results

### 3.1 Wheat yield and rhizosphere soil biological properties

**Figure 1:**
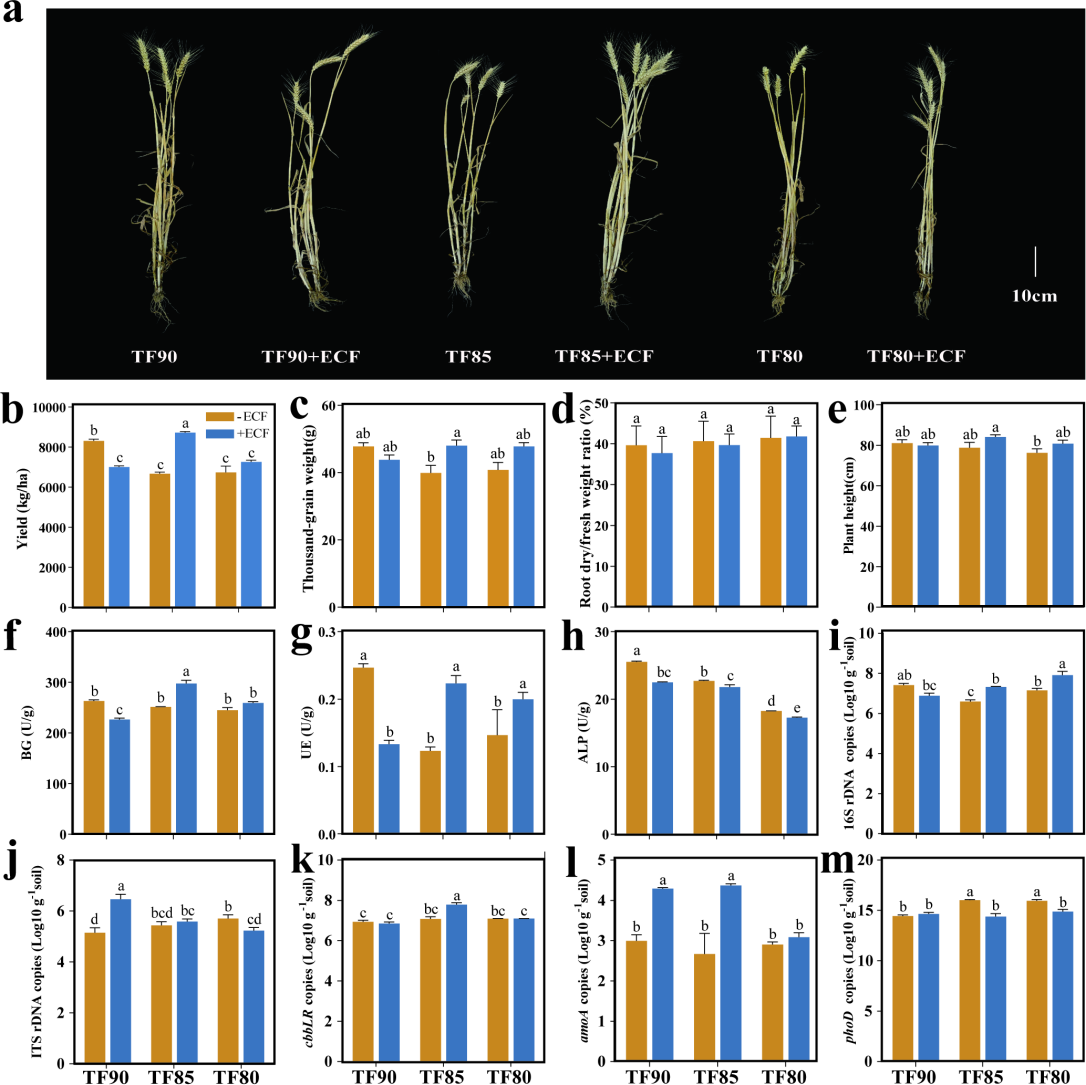
Effects of fertilizer reduction combined with ecological composite fertilizer (ECF) application on wheat yield and rhizosphere soil properties. **(a)** Phenotypic images of wheat plants at maturity period; **(b–e)** Yield, thousand-grain weight, root dry/fresh weight ratio, and plant height at maturity period; **(f–h)** Soil extracellular enzyme activities. **(f)** β-1,4-glucosidase (BG), **(g)** urease (UE), and **(h)** alkaline phosphatase (ALP); **(i)** Bacterial 16S rRNA gene copy number. **(j)** Fungal ITS gene copy number. **(k)** Relative abundance of the *cbbL* gene (carbon cycling). **(l)** Relative abundance of the *amoA* gene (nitrogen cycling). (m) Relative abundance of the *phoD* gene (phosphorus cycling). Data represent mean ± SD (n = 3). Different superscript letters denote statistically significant differences among treatments (*P* < 0.05).

We assessed wheat agronomic performance at the jointing, flowering, and maturity periods under gradient fertilization with or without ECF (**Figure 1**, **Supplementary Figure S2**). At the jointing period, no significant differences were observed among treatments (**Supplementary Figures S2a–d**). During flowering, the TF85+ECF group produced a higher spike number than TF85 (**Supplementary Figures S2e–i**) (*P* < 0.05). Pronounced differences emerged at maturity period, the yield of TF85+ECF group was significantly greater than that of in all other groups (**Figures 1a, b**) (*P* < 0.05). The thousand grain weight (TGW) increased from 39.9 g in TF85 to 48.0 g in the TF85+ECF group (**Figure 1c**) (P < 0.01). Notably, wheat yield reached its maximum in the TF85+ECF group (8,717.33 kg/ha), representing a 30.63% increase over TF85. Structures of AMF colonization, which included arbuscules, vesicles, and hyphae, were observed in wheat roots across all six treatments (**Supplementary Figure S2l**). In the -ECF treatment groups (TF90, TF85, TF80), colonization rates averaged 68.74% (TF90), 59.38% (TF85), and 44.79% (TF80). The +ECF treatment groups averaged 36.46% (TF90+ECF), 70.95% (TF85+ECF), and 67.41% (TF80+ECF). Under 10% fertilizer reduction, the colonization rate in the TF90+ECF group was significantly lower than in the TF90 group (*P* < 0.01). In contrast, under 15% reduction, the TF85+ECF group maintained a high colonization rate comparable to the TF90 group. AMF spore density was higher in +ECF treatment groups than in the corresponding -ECF treatment groups at equivalent fertilization levels (**Supplementary Figures S2m, n**). The maximum density (120 spores per 15 g soil) was recorded in the TF85+ECF group, followed by the TF85 group (87 spores).

Analysis of soil extracellular enzyme activity revealed differential effects of ECF co-application depending on the fertilization level (**Figures 1f–h**). The TF90+ECF group significantly reduced the activities of BG, UE, and ALP compared to its control without ECF (*P* < 0.05). In contrast, the TF85+ECF group significantly increased BG and UE activities but decreased ALP activity (*P* < 0.05). Both the TF85+ECF and TF80+ECF groups exhibited a similar response pattern relative to their controls. We quantified the copy numbers of bacterial 16S rRNA genes, fungal internal transcribed spacer (ITS) regions, and key functional genes involved in carbon (*cbbLR*), nitrogen (*amoA*), and phosphorus (*phoD*) cycling across different soil treatment groups (**Figures 1i–m**). Under identical fertilization regimes, bacterial 16S rRNA gene copy numbers were significantly elevated in the TF85+ECF and TF80+ECF groups compared to the control (*P <* 0.01), whereas the TF90+ECF group showed no significant change. Fungal ITS copy numbers were significantly lower across all treatments relative to the TF90+ECF (*P <* 0.05). With the exception of the non-significant difference between the TF85+ECF and the TF85 groups, fungal ITS levels differed significantly among the remaining treatments (*P* < 0.05). Among the functional genes, *cbbLR* copy numbers peaked in the TF85+ECF group (*P* < 0.001). *amoA* gene abundance increased significantly in the TF90+ECF and TF85+ECF groups relative to the control (*P <* 0.01 and *P <* 0.05, respectively), while *phoD* gene levels were significantly reduced in the TF85+ECF and TF80+ECF groups (*P <* 0.01, *P <* 0.05).

### 3.2 Diversity patterns in soil microbial communities

**Figure 2:**
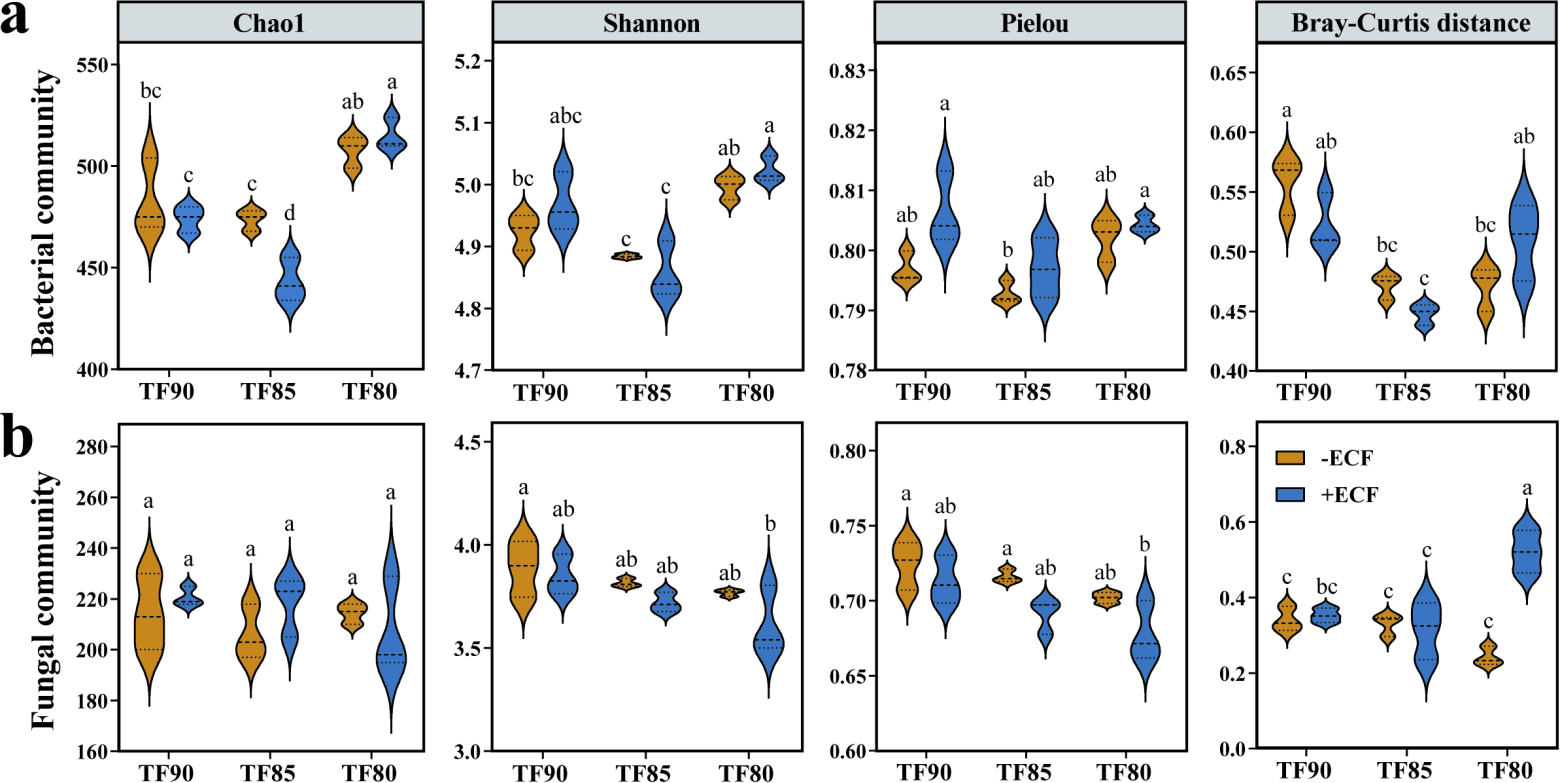
Alpha and beta diversity of bacterial and fungal communities in six treatments. a and b represent violin plots of the Chao1, Shannon, Pielou indexes and Bray-Curtis distance in bacterial **(a)** and fungal **(b)** communities. Data represent mean ± SD (n = 3). Different superscript letters denote statistically significant differences among treatments (*P* < 0.05).

To assess the effects of co-applying ECF under different fertiliser reduction treatments on rhizosphere soil microbial diversity, we analyzed the Chao1 (abundance), Shannon (diversity), and Pielou (evenness) indices for bacterial and fungal communities (**Figure 2**). For bacterial communities, the Chao1 index in the TF80+ECF group was significantly higher than that in the TF90+ECF and TF85+ECF groups (*p <* 0.05). At the same fertilization level, the TF85+ECF group exhibited the lowest richness, with a significantly lower Chao1 index compared to the TF85 control group and the other treatments (*p* < 0.05) (**Figure 2a**). Analysis of the Shannon index showed no significant differences among the TF90+ECF, TF85+ECF, and TF80+ECF groups relative to the control; however, the TF85+ECF group was significantly lower than the TF80+ECF group (*p* < 0.001). Although Pielou evenness showed no significant differences between +ECF and control groups (*p* > 0.05), it was slightly higher in the +ECF treatment groups (**Figure 2a**).

For fungal communities, the Chao1, Shannon, and Pielou indices showed no significant differences between the +ECF and -ECF treatment groups (*p* > 0.05), and both groups exhibited highly similar distribution patterns (**Figure 2b**). To assess community structure, beta diversity was analyzed based on Bray-Curtis distances. Within bacterial communities, the TF90, TF85, and TF80 groups showed no significant differences before and after ECF application. In contrast, the TF85+ECF group exhibited significant differences in community structure compared to both the TF90+ECF and TF80+ECF groups (*p <* 0.01, *p <* 0.05). In fungal communities, the Bray-Curtis distance for the TF80+ECF group was significantly higher than the control group. Furthermore, within the +ECF treatment groups, the TF80+ECF group also significantly exceeded the TF90+ECF and TF85+ECF groups (**Figure 2b**).

### 3.3 Composition of the wheat rhizosphere microbial community

**Figure 3:**
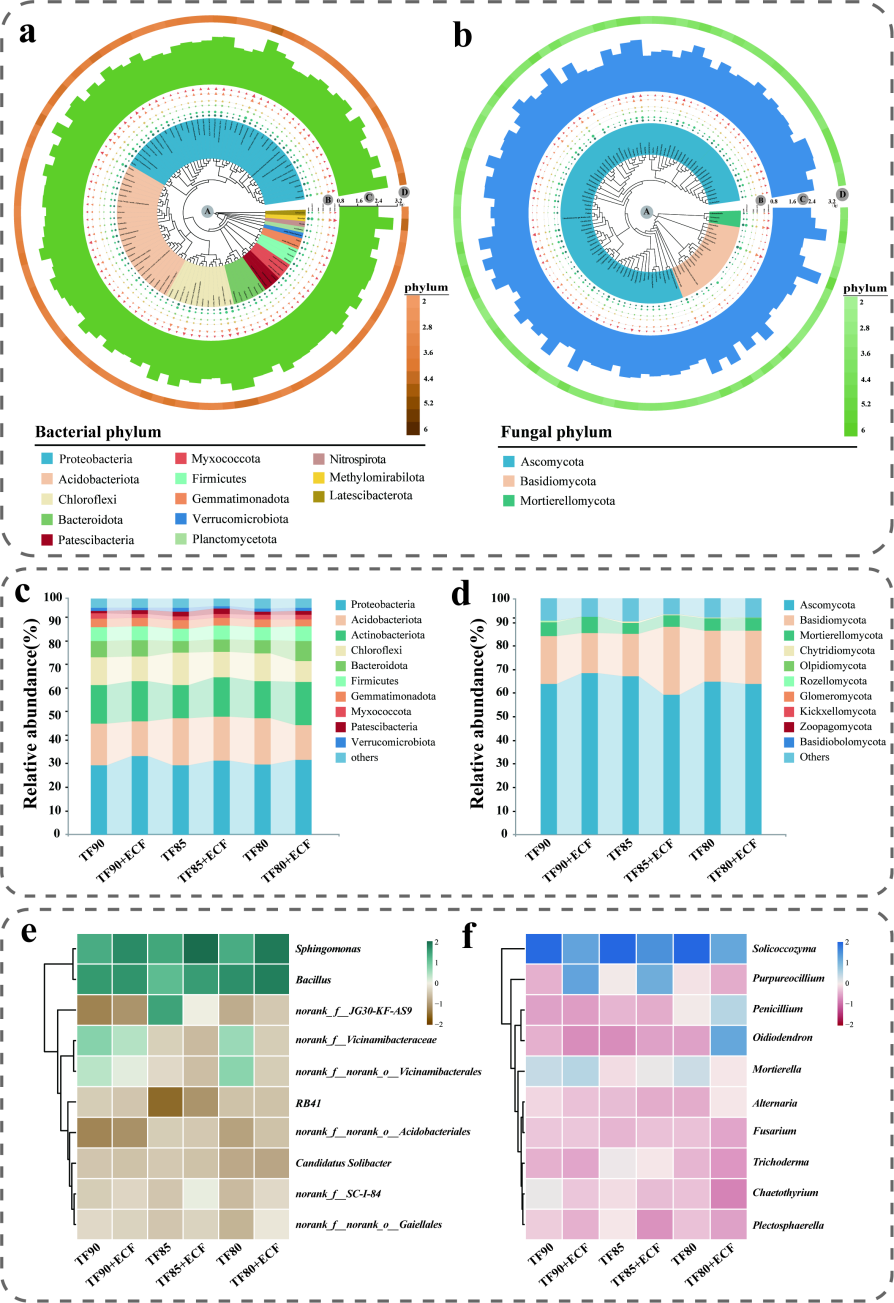
Composition of the wheat rhizosphere microbial community. Phylogenetic trees of the top 100 bacterial **(a)** and fungal **(b)** genera across six treatments. **(A)** Phylogenetic reconstruction (MEGA-X) with terminal branches colored by phylum assignment. **(B)** Read abundance per genus across samples; **(C)** Total read abundance of the top 100 genera across all samples; **(D)** Phylum level abundance composition within the top 100 genera. All data were natural logarithm (log10) transformed prior to visualization. Phylum level distribution of the top 10 soil bacterial **(c)** and fungal **(d)** communities under different treatments. Genus level abundance heatmaps of the top 10 rhizosphere bacterial **(e)** and fungal **(f)** communities.

To characterize microbial community composition and relative abundance variations across samples, we analyzed the top 10 phyla and genera (**Figures 3c–f**). The phylum level community profiling revealed consistent pattern, bacterial communities across all samples were dominated by Proteobacteria, Acidobacteriota, Actinobacteriota, Chloroflexi, and Bacteroidota (collectively >80% relative abundance; **Figure 3c**). Fungal communities primarily comprised Ascomycota, Basidiomycota, and Mortierellomycota, where Ascomycota and Basidiomycota exhibited absolute dominance (collectively accounting for >84% relative abundance; **Figure 3d**). Notably, the relative abundance of Basidiomycota exhibited no significant variation across the three -ECF control groups (**Figures 3e, f**). In contrast, within +ECF treatment groups, the TF85+ECF group displayed significantly higher Basidiomycota abundance than the TF90+ECF and TF80+ECF groups (*p <* 0.05). Heatmap analysis of the top 10 genera by relative abundance (**Figures 3e, f**) revealed that the bacterial core genera *Sphingomonas* and *Bacillus* maintained stable relative abundances across treatments. Conversely, the fungal genus level community structure showed significant differentiation. Specifically, *Purpureocillium* abundance was significantly elevated in the TF90+ECF and TF85+ECF groups than in the TF80+ECF group (*p <* 0.05), while *Trichoderma* showed significantly higher abundance in the TF85+ECF group than in the TF90+ECF and TF80+ECF groups (*p <* 0.05). Overall, the fungal community exhibited more pronounced structural shifts in response to ECF application under gradient fertilizer reduction compared to the bacterial community.

### 3.4 Identify keystone taxa of microbial communities

**Figure 4:**
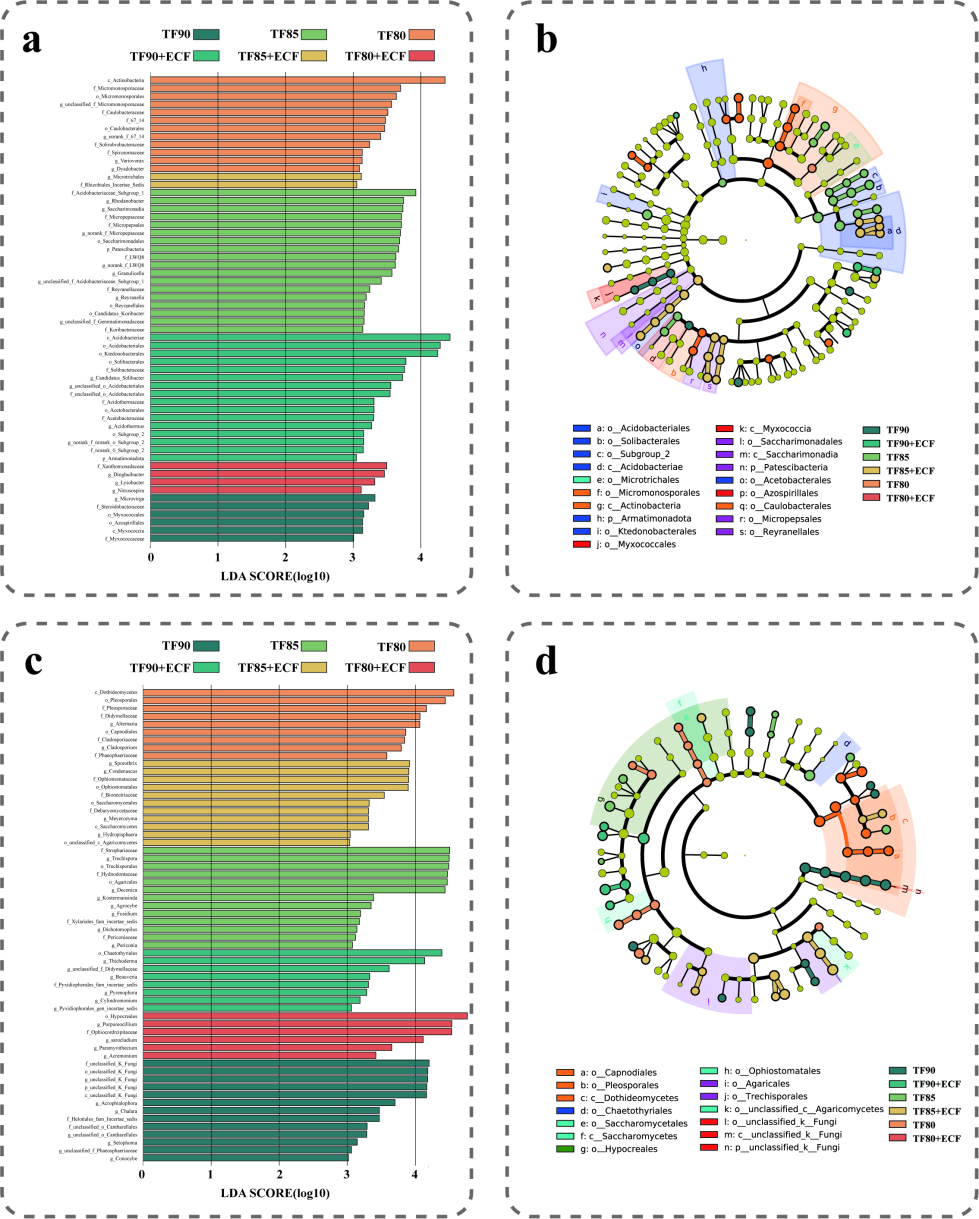
Linear discriminant analysis (LDA) effect size (LEfSe) (LDA score threshold ≥3) shows the phylogenetic distribution of microorganisms in the six treatments. Taxa from phylum to genus levels are shown: **(a, b)** Bacteria and **(c, d)** Fungi.

To identify key microbial taxa influenced by gradient fertiliser reduction and ECF application in the wheat rhizosphere, we performed LEfSe analysis to compare core microbial groups showing significant abundance variations among treatment groups (**Figure 4**). The analysis was conducted using a LDA score threshold of 3.0 (**Figures 4a, c**) and a Kruskal Wallis test p value of 0.05(**Figures 4b, d**). For bacteria, a total of 60 significant biomarkers were identified among the six treatment groups, distributed as follows: TF90 (6), TF90+ECF (16), TF85 (18), TF85+ECF (2), TF80 (14), and TF80+ECF (4). Notably, under 15% and 20% fertilizer reduction, the numbers of bacterial biomarkers in control and treatment groups showed trends opposite to those observed under the 10% reduction. In +ECF treatment groups, the TF85+ECF and TF80+ECF groups exhibited reduced numbers of bacterial biomarkers, with significant enrichment of beneficial or neutral taxa. Specifically, the TF85+ECF group was enriched with *g_Microtrichales* and f_Rhizobiales_Incertae_Sedis, while the TF80+ECF group was enriched with f_Xanthomonadaceae, *g_Dinghuibacter*, *g_Lysobacter*, and *g_Nitrosospira*.

For fungi, 60 significant biomarkers were identified across treatments: TF90 (13), TF90+ECF (8), TF85 (13), TF85+ECF (11), TF80 (9), and TF80+ECF (6). In -ECF groups, TF90 exhibited a disturbed community structure, characterized by a high proportion of unclassified fungal units; TF85 was enriched with saprotrophic fungi such as Agaricales; and TF80 was significantly enriched with potential pathogenic taxa, including Alternaria and Cladosporium. In contrast, ECF application led to the enrichment of stress tolerant and defensive taxa in the TF90+ECF group, including Chaetothyriales and *Trichoderma*. The TF80+ECF group recruited biocontrol related fungi within Hypocreales, such as Purpureocillium and Acremonium. The TF85+ECF group specifically enriched the following fungal taxa: strong organic matter decomposers (e.g., the order Ophiostomatales and unclassified Agaricomycetes), rapid carbon cycling groups (e.g., the order Saccharomycetales and the genus Meyerozyma), as well as taxa with biocontrol potential (e.g., the family Bionectriaceae).

**Figure 5:**
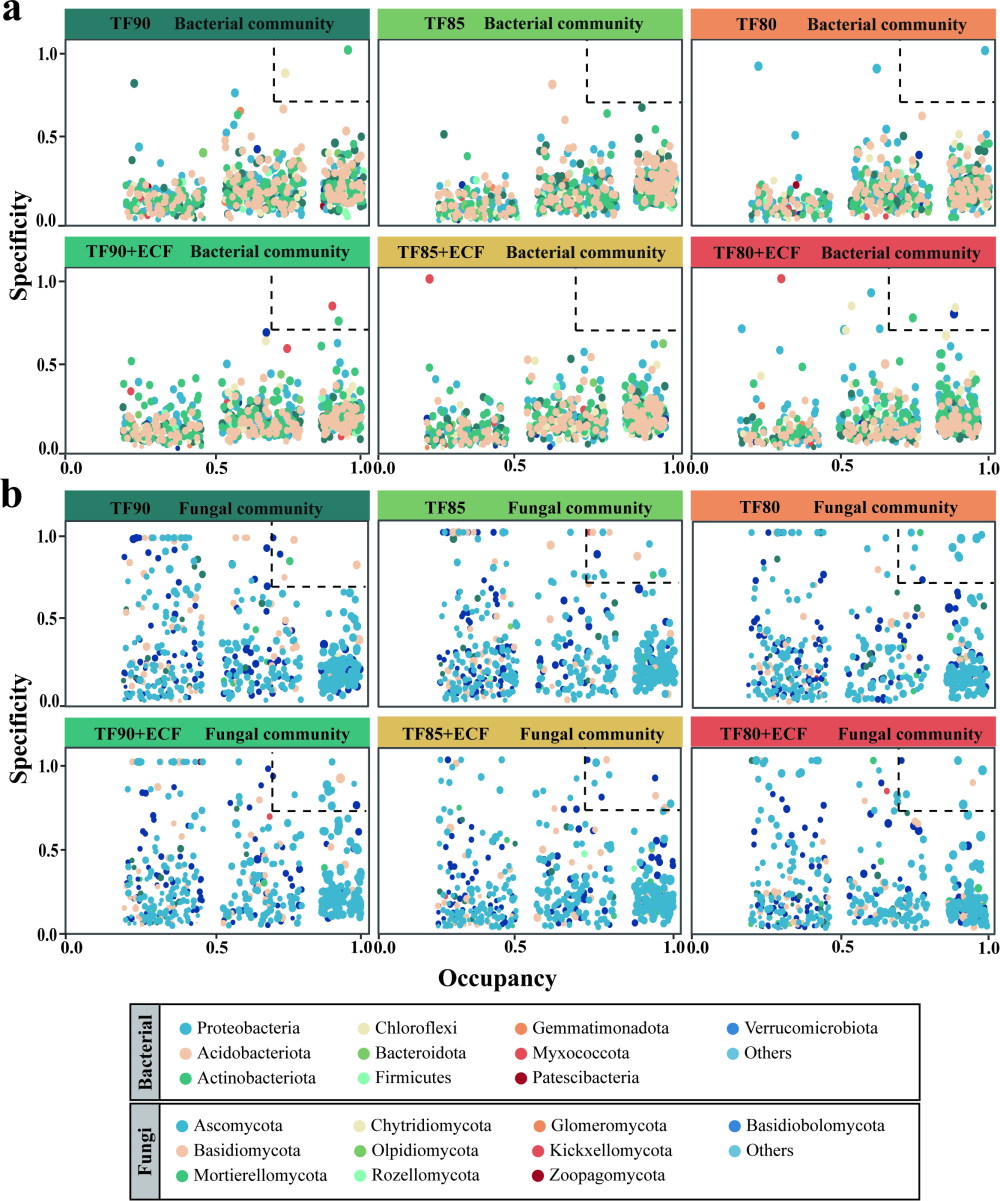
The specialization-occupancy (SPEC-OCCU) profiles of habitat-specific communities. **(a)** Bacterial and **(b)** fungal communities are shown separately. Plots are based on the top 1000 abundant amplicon sequence variants (ASVs) per habitat; the x-axis represents occupancy (distribution breadth across sites) and the y-axis the specialization index. Points are sized by mean relative abundance and colored by phylum; the dashed line indicates the neutral model expectation.

To investigate the distribution patterns and habitat specificity of ASVs across different treatment groups, we calculated the specificity and occupancy rate for each ASV (**Figure 5**). SPEC-OCCU analysis revealed that in the bacterial communities (**Figure 5a**), four key ASVs were identified in the -ECF treatment groups, classified under the phyla Actinobacteriota, Chloroflexi, and Proteobacteria. Conversely, seven key ASVs were identified in the +ECF treatment groups, belonging to the phyla Proteobacteria, Patescibacteria, Actinobacteriota, and Bacteroidota. Within the fungal communities, 23 keystone ASVs were identified in the -ECF treatment groups. In contrast, the +ECF treatment groups contained 37, representing a significant increase over the non-inoculated control (**Figure 5b**).

### 3.5 Co-occurrence networks of bacterial and fungal communities

**Figure 6:**
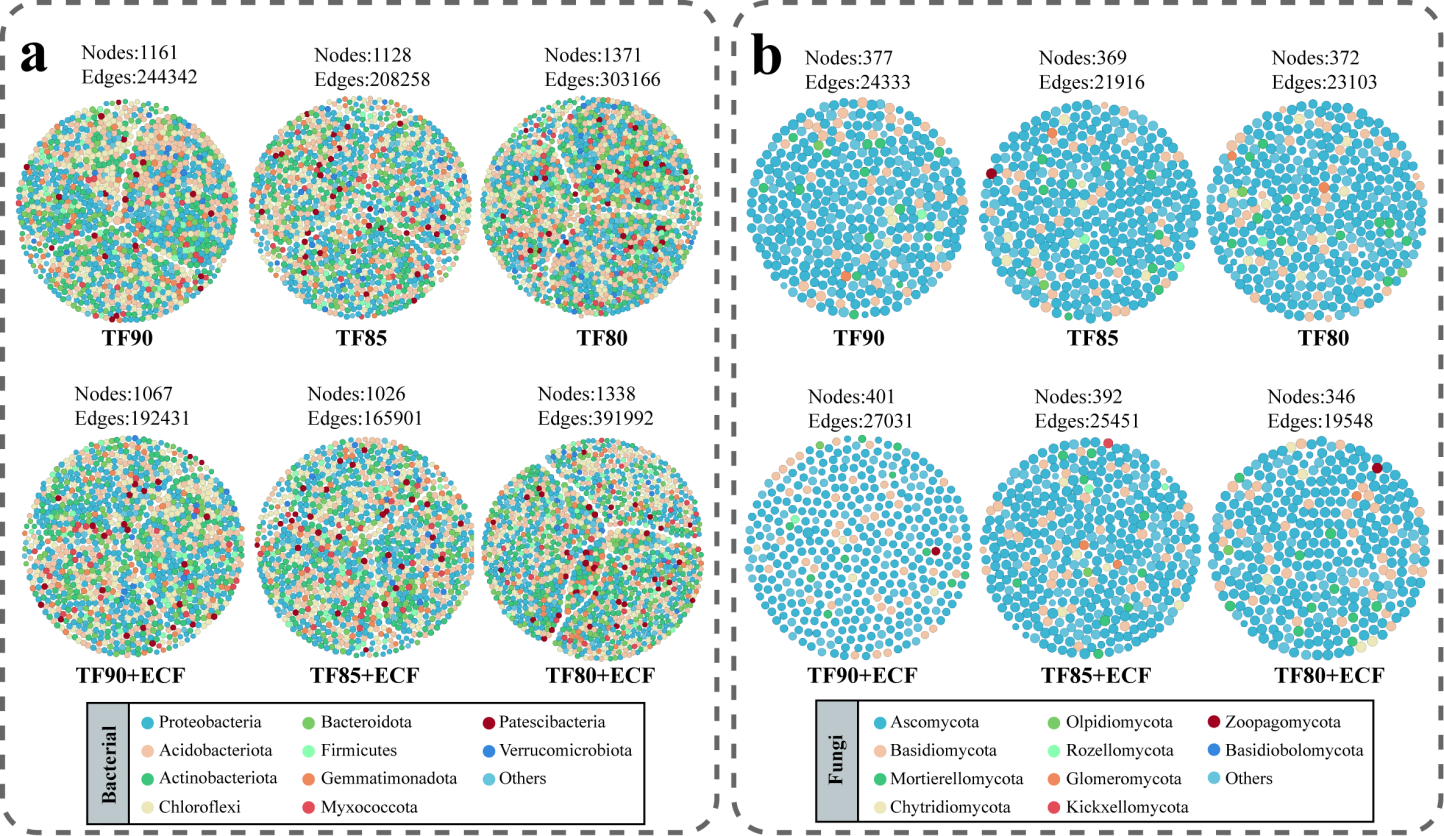
Co-occurrence networks of bacterial and fungal communities. **(a)** Bacterial network; **(b)** Fungal network. The size of each node corresponds to the degree (number of connections) within the network, and each node is colored by phylum.

Network analysis revealed the impact of reduced fertiliser application combined with ECF on the ecological niche structure of the rhizosphere microbial community and was used to assess the complexity and connectivity of interspecific interactions under different treatments (**Figure 6**). The co-abundance networks of bacteria and fungi were constructed using Random Matrix Theory (RMT) with a similarity threshold set at 0.75.

In the bacterial networks (**Figure 6a**), nodes were predominantly affiliated with the phyla Proteobacteria, Acidobacteria, Chloroflexi, and Actinobacteria. Under reduced fertiliser conditions, the TF85 treatment showed the lowest number of nodes and edges. Application of ECF generally maintained the overall trends among treatments. At a given fertilization level, the +ECF treatment groups consistently exhibited reduced node counts compared to the -ECF groups, indicating a decrease in bacterial diversity and network complexity. Total connections across treatments ranged from 165,901 to 391,992, demonstrating numerous microbial interactions within the networks. Specifically, in the TF90+ECF and TF85+ECF groups, the combination of fertilizer reduction with ECF resulted in a decrease in both node number and connectivity. The proportion of positive correlations ranged from 46.1% to 48.53%, while negative correlations accounted for 51.47% to 53.9%, indicating a balanced profile of cooperative and competitive interactions without a dominant type.

In the fungal networks (**Figure 6b**), nodes were mainly composed of Ascomycota and Basidiomycota. Node counts varied between 346 and 401, reflecting differences in network size across treatments. The number of edges ranged from 14,384 to 27,031, underscoring substantial microbial interplay. With fertiliser reduction, fungal node and edge counts initially increased and then decreased, reaching minima in the TF85 treatment. ECF application reinforced this trend: the +ECF treatment groups showed increased node and edge numbers compared to their fertiliser reduced counterparts (TF90 vs TF90+ECF, TF85 vs TF85+ECF), with the TF85+ECF group exhibiting notably enhanced network complexity. Positive correlations constituted 45.75%-48.75% of edges, and negative correlations 51.25%-54.25%. The TF90+ECF and TF85+ECF groups exhibited a greater number of positive connections compared to their respective controls, an outcome associated with the application of ECF.

### 3.6 Relationships between environmental factors and microbial community structure

**Figure 7:**
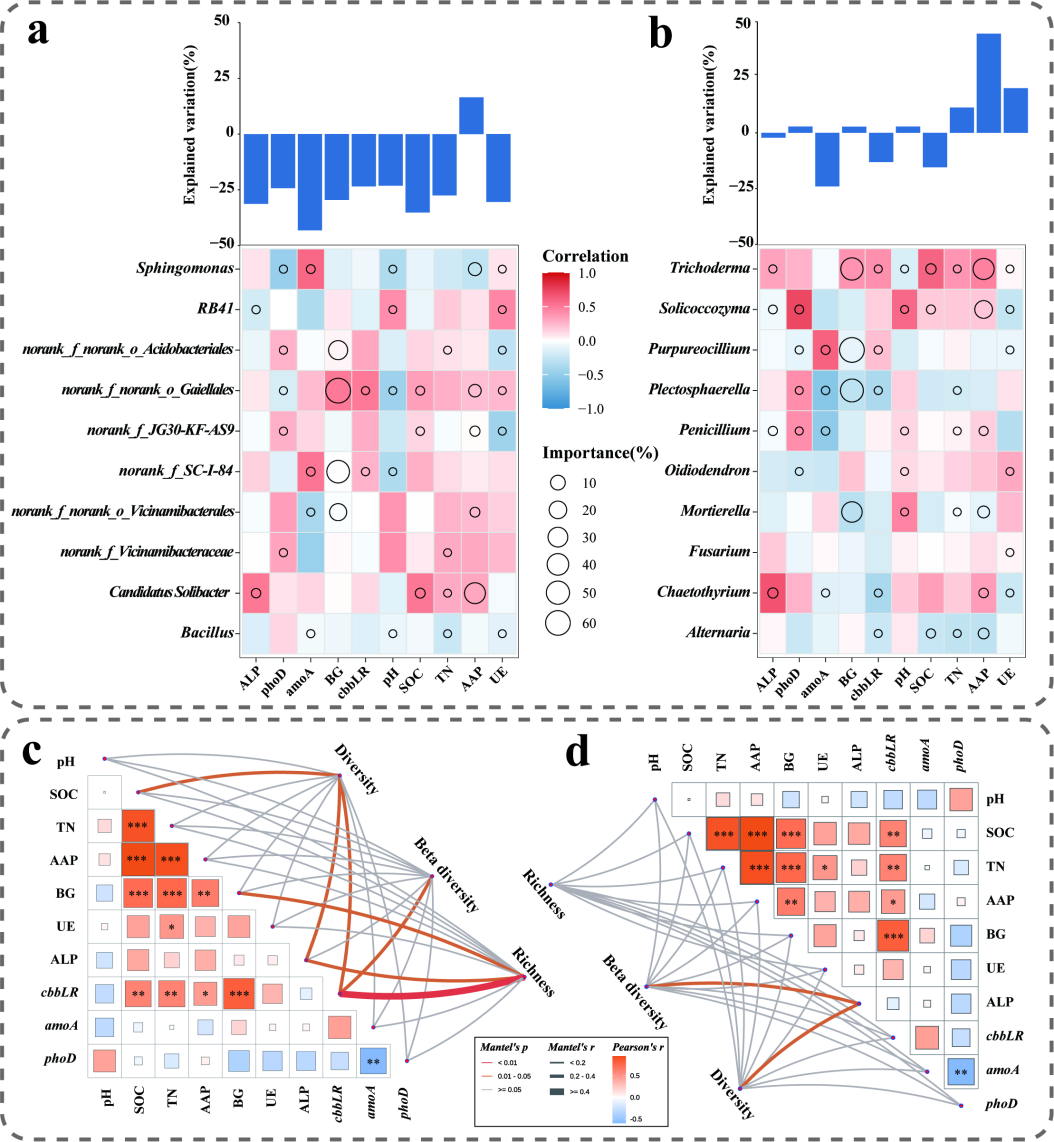
Relationships between environmental factors and microbial community structure. Association between specific ecosystem functionalities and **(a)** bacterial communities or **(b)** fungal communities, analyzed via random forest regression and Spearman correlation based on the top 10 most abundant genera. Circle size represents variable importance, and color indicates the Spearman correlation coefficient. **(c)** Mantel test correlation network between soil enzyme activities (β-1,4-glucosidase, BG; urease, UE; alkaline phosphatase, ALP), physicochemical properties (pH), key soil nutrient indicators (soil organic carbon, SOC; total nitrogen, TN; available phosphorus, AAP), C-, N-, P-cycling functional genes (*cbbLR*; *amoA*; *phoD*), and bacterial diversity (represented by the Chao1 index for richness, the Shannon index for diversity, and the Bray-Curtis distance for beta diversity). **(d)** Corresponding network for fungal diversity. The width and the color of the lines show the Mantel test’s R value and P value, respectively.

We further examined associations between microbial composition and specific ecosystem functions through correlation analyses (**Figures 7a, b**). In bacterial communities, BG activity, *cbbLR* gene abundance, and SOC content showed significant positive correlations with overall community structure. At the genus level, *g:norank_f:Vicinamibacteraceae* and *Candidatus_Solibacter* were strongly positively correlated with ecosystem functions, while Bacillus correlated negatively. More pronounced correlations were observed in fungal communities, *Solicoccozyma* and *Trichoderma* exhibited positive associations with multifunctionality, whereas Alternaria was negatively correlated. Notably, BG activity correlated positively with *Trichoderma* but negatively with *Purpureocillium*, *Plectosphaerella*, and *Mortierella*.

To assess the linkage between microbial functional profiles and community diversity, we performed Mantel tests based on alpha and beta diversity metrics (**Figures 7c, d**). The results indicated that SOC, BG, ALP, and *cbbLR* were significantly correlated with both the alpha diversity (assessed via the Chao1 richness index and the Shannon diversity index) and beta diversity (Bray-Curtis distance) of bacterial communities (Mantel’s r > 0.25, *P* < 0.05). For fungal communities, ALP was significantly correlated with both the Shannon index and Bray-Curtis distance (Mantel’s r > 0.25, *P* < 0.05). To investigate the linkage between functional composition profiles and diversity of soil microbial communities, we performed Mantel tests incorporating functional Chao1 and Shannon indices, as well as Bray-Curtis distances. The results indicated that SOC, BG, ALP, and *cbbLR* were significantly correlated with both alpha and beta diversity of bacterial communities (Mantel’s r > 0.25, *P <* 0.05). Within fungal communities, ALP also showed significant correlations with the Shannon index and Bray-Curtis distance (Mantel’s r > 0.25, *P <* 0.05).

## 4 Discussion

### 4.1 Effects of fertilizer reduction and ECF application on wheat growth

In addressing the challenges of resource waste, environmental pollution, and soil degradation stemming from excessive chemical fertilizer application, eco-friendly and sustainable approaches typified by AMF provide a pivotal pathway for promoting agricultural sustainability (Sun and Shahrajabian, 2023; Li et al., 2025). In field experiment, the TF85+ECF group characterized by AMF and BC application combined with a 15% reduction in fertilizer achieved the highest wheat yield among all tested treatments (**Figure 1**). The synergistic effect of AMF and BC enhanced plant performance. BC amendment improved key soil physicochemical properties and provided a more favorable microenvironment for AMF colonization (Nirukshan et al., 2022). Concurrently, successful AMF root colonization led to considerable hyphal expansion, which significantly broadened the nutrient absorption range and improved acquisition efficiency (Yuan et al., 2025). These findings collectively support the feasibility and effectiveness of the AM and biochar co-application as a sustainable wheat production strategy (Zhao et al., 2024). Notably, under a 10% fertilizer reduction (TF90+ECF), the symbiotic relationship between AMF and the host plant was not significantly strengthened, likely due to the relatively sufficient soil nutrient levels, which reduced the plant’s dependency on mycorrhizal facilitation. In contrast, when fertilizer input was reduced by 15% and 20%, both root colonization intensity and arbuscular abundance of AMF were markedly increased (**Figure 1**). Consistent with previous findings, under nutrient-limited conditions, plants tend to establish tight symbiotic relationships with (AMF) as an adaptive strategy to mitigate nutrient stress (Basiru et al., 2023). This finding further underscores that a moderate reduction in fertilizer application can actively stimulate the formation and functioning of the plant-AMF symbiotic system (Nirukshan et al., 2022). In short, under a 15% fertilizer reduction combined with ECF, the symbiotic interaction between AMF and wheat was significantly enhanced, effectively increasing grain yield. This combination represented the optimal treatment for maintaining stable yield with reduced fertilizer input in this trial.

### 4.2 Effects of fertiliser reduction combined with ECF application on microbial communities

As fundamental yet highly complex structural components of ecosystems, soil microorganisms drive nutrient cycling and energy flux across the plant-soil-atmosphere continuum (Brüggemann et al., 2011). In our study, the combined application of fertiliser reduction and ECF led to discernible shifts in microbial community diversity and composition (**Figures 2**, **3**). Analysis of community diversity indicated that bacterial communities were particularly sensitive to these treatments. The TF85+ECF group exhibited a significant reduction in bacterial richness, while the TF80+ECF group showed the most substantial impact on fungal community structure (**Figure 2**). These patterns may reflect adaptive responses of the plant microbiome to altered soil conditions especially under nutrient stress (Bulgarelli et al., 2022). Notably, the introduction of ECF likely modified the composition of root exudates, enriching specific rhizosphere-associated microorganisms and contributing to the observed divergence in beta diversity among soil samples across treatments (Huang et al., 2024).

Phylogenetic tree analysis revealed compositional differences in the core bacterial and fungal communities among the six treatment groups (**Figure 3**). Under varying fertiliser reduction levels combined with ECF application, clear ecological succession patterns were observed in both bacterial and fungal communities. At the phylum level, the wheat rhizosphere was dominated by Proteobacteria, Actinobacteria, Acidobacteria, and Chloroflexi, consistent with previous findings in agricultural soils (Basiru et al., 2023). The addition of ECF led to an increase in the relative abundance of Proteobacteria and Actinobacteria, while Acidobacteria declined. This shift may be attributed to AMF mediated competition for carbon sources in the rhizosphere (Weber et al., 2025). Notably, certain proteobacteria members, capable of utilizing root exuded carbon for growth promotion, have been shown to enrich significantly on AMF hyphal surfaces (Emmett et al., 2021). The decline in Acidobacteria, which typically thrive in oligotrophic conditions, may reflect resource competition with AMF, whereas Actinobacteria may benefit from synergistic interactions with the fungus, supporting their increased abundance (Cheng et al., 2012; Kaiser et al., 2015). At the genus level, *Sphingomonas* and *Bacillus* were identified as the dominant taxa. The introduction of AMF is likely to improve the soil microenvironment and stimulate root exudation, thereby supplying more carbon sources and expanding ecological niches for bacteria such as *Sphingomonas* (Kaiser et al., 2015; Zhou et al., 2020). Previous studies have recognized several rhizobacterial genera, including *Bacillus*, *Rhizobium*, *Arthrobacter*, *Pseudomonas*, and *Agrobacterium*, serving as plant growth-promoting rhizobacteria (PGPR) (Compant et al., 2019). These microorganisms are not only widely applied in agricultural systems for their beneficial traits but also enhance plant resilience under diverse environmental stresses, thereby supporting overall plant health and productivity (Ilangumaran and Smith, 2017; Fitzpatrick et al., 2018). More importantly, co-occurrence network analysis revealed that these compositional changes underpinned a fundamental reorganization of microbial interactions (**Figure 6**). While AMF inoculation simplified bacterial network architecture-suggesting a selective filtering effect-it significantly enhanced the complexity of the fungal ecological network, particularly under the TF85+ECF treatment.

The dynamic equilibrium of plant rhizosphere microbiota plays a fundamental role in sustaining plant growth and health (Trivedi et al., 2020). In our study, integrated LEfSe and SPEC-OCCU analyses revealed the synergistic regulatory effects of gradient fertilizer reduction combined with ECF on the wheat rhizosphere microbial community (**Figure 4**, **Figure 5**). Under moderate fertilizer reduction (TF85+ECF), fungal communities were enriched with taxa associated with diverse ecological functions, including strong decomposers (Ophiostomatales), rapid nutrient cyclers (Rhodophorales), and documented biocontrol agents (Bionectriaceae). This indicates a potent steering effect of AMF toward multifunctional fungal assemblages under moderate nutrient stress (Wang et al., 2017). In contrast, under severe nutrient depletion (TF80+ECF), the fungal community shifted toward a functional structure dominated by stress tolerant and biocontrol lineages, such as Hypholomorpha and Rhizopus (Lin et al., 2015). Nutrient gradient driven succession highlights soil nutrient availability as a critical boundary condition regulating the outcome of AMF-fungal interactions (Johnson, 2010). Compared with the -ECF control groups, the +ECF treatment groups systematically enriched fungal taxa with clearly defined biocontrol functions, such as *Trichoderma* and *Paecilomyces*. These results demonstrate the pivotal role for ECF in steering the rhizosphere fungal community toward a disease suppressive state, even under varying nutrient regimes. The biochar component likely shapes the community by altering the physico-chemical niche (e.g., pH, porosity, and nutrient adsorption), which can favor oligotrophic bacteria such as *Candidatus_Solibacter*. Concurrently, the AMF component may directly or indirectly recruit beneficial fungi like *Trichoderma* and *Solicoccozyma*, potentially via modulation of root exudate profiles or the creation of hyphal-based colonization pathways. This synergy creates a composite habitat filter that shapes the keystone microbiome structure under ECF treatment.

Network analysis further elucidated the synergistic regulatory mechanisms of fertilizer reduction and ECF on rhizosphere microbial communities at the level of ecological interactions (**Figure 6**). Notably, bacterial and fungal networks exhibited fundamentally distinct response patterns to identical treatment strategies (Barberán et al., 2012). The introduction of AMF generally simplified bacterial network architecture, as evidenced by reduced node numbers and connectivity. The streamlining suggests that AMF may exert a selective filtering effect on certain bacterial taxa through resource competition or niche modification, thereby refining bacterial interaction networks (Shi et al., 2020). In stark contrast, fungal networks displayed significantly enhanced complexity following ECF application, particularly under moderate nutrient reduction (TF85+ECF), where both node abundance and connection density markedly increased. This structural enhancement likely reflects the distinctive ecological strategies of fungi, while bacteria respond rapidly to nutrient shifts through population dynamics, fungi rely more extensively on the physical architecture of mycelial networks and their spatio temporal resource allocation capabilities (Wagg et al., 2019; Shu et al., 2022). Crucially, the observed augmentation of fungal network complexity in the TF85+ECF treatment aligns precisely with LEfSe results indicating the concurrent enrichment of functionally complementary fungal groups-including efficient decomposers, nutrient cyclers, and documented biocontrol agents (**Figure 4**, **6**) (Toju et al., 2018). The convergence of network topology and taxonomic composition suggests that moderate nutrient stress, when coupled with AMF inoculation, promotes the assembly of a more interconnected and functionally integrated fungal community (Shu et al., 2022).

### 4.3 Microbial functional stratification and keystone taxa drive soil nutrient cycling functions under fertilizer reduction and ECF application

Further investigation through functional gene quantification and multivariate analysis uncovered the microbial functional basis for the synergistic effects of fertilizer reduction and ECF application at the soil ecosystem level (**Figures 1**, **7**). The TF85+ECF group elicited the most coherent functional response, marked by a peak in the copy number of the key carbon fixation gene *cbbLR* and a significant increase in bacterial 16S rRNA gene abundance, indicating effective activation of the rhizosphere bacterial community’s carbon sequestration potential under moderate fertilizer reduction (Yuan et al., 2012). This response was further corroborated by the concurrent elevation in BG and UE activities, reflecting enhanced internal carbon and nitrogen cycling to compensate for the reduced external fertilizer input. In contrast, the TF90+ECF group led to a decline in extracellular enzyme activities (BG, UE, ALP), suggesting that ample nutrient availability downregulated microbial investment in resource acquisition in favor of growth and maintenance (Mooshammer et al., 2014; Kuypers et al., 2018). Under more severe fertilizer reduction (TF80+ECF), although bacterial abundance and BG/UE activities increased, the functional response was less pronounced than in TF85+ECF group, underscoring the context dependent efficacy of ECF. Regarding nutrient cycling genes, *amoA* increased significantly in both the TF90+ECF and TF85+ECF groups, pointing to stimulated ammonia oxidation under these regimes (Ouyang et al., 2016). Conversely, the phosphorus cycling gene *phoD* was suppressed under the TF85+ECF and TF80+ECF treatments, revealing differential sensitivity of nutrient cycles to management intensity (Fraser et al., 2015). Mantel tests further established that SOC, BG activity, and *cbbLR* gene abundance were not merely biochemical outcomes but also key environmental drivers significantly correlated with bacterial alpha and beta diversity, reinforcing the mechanistic link between functional shifts and community structure. Collectively, these findings demonstrate that nutrient availability acts as a primary filter shaping microbial metabolic priorities, leading to a functional stratification wherein carbon and nitrogen cycling are promoted under moderate fertilizer reduction combined with ECF (Mooshammer et al., 2014; Kuypers et al., 2018).

By integrating functional gene profiles with microbial community composition, we identified specific microbial taxa closely linked to soil microbial functional potential (**Figures 3**, **7**). Within bacterial communities, genera including *g_norank_f:Vicinamibacteraceae* and *Candidatus_Solibacter* demonstrated significant positive correlations with multifunctionality, whereas *Bacillus* showed a negative association. *Candidatus_Solibacter* belong to the phylum Acidobacteria. Previous studies have demonstrated that acidobacterial taxa possess a broad suite of functional genes involved in carbon, nitrogen, and sulfur cycling, including those responsible for degrading complex polysaccharides, encoding nutrient transporters, and regulating secondary metabolite synthesis (Wang et al., 2024). These functional attributes position them as crucial drivers of organic matter transformation and nutrient cycling in soil ecosystems. More pronounced correlation patterns emerged in fungal communities, *Trichoderma*-known for its biocontrol capabilities, and the metabolically versatile *Solicoccozyma* both exhibited positive correlations with multifunctionality, in contrast to the potential pathogen, *Alternari* displayed negative correlations (Song et al., 2023; Woo et al., 2023). These findings not only corroborate the soil ecological significance of enhanced fungal network complexity observed in the TF85+ECF group from our network analyses(**Figures 6**), but further pinpoint the functional importance of specific keystone taxa in maintaining rhizosphere community stability and multifunctionality (Banerjee et al., 2018; Wagg et al., 2019). The coordinated presence of these beneficial taxa appears to establish a synergistic framework that supports both microbial ecosystem functioning and plant growth, providing a mechanistic explanation for the improved plant performance under combined fertilizer reduction and ECF application (Toju et al., 2018).

In synthesis, a 15% fertilizer reduction combined with ECF initiates a cascade of beneficial rhizosphere microbial assembly processes, ultimately sustaining crop yield stability and enhancing soil nutrient cycling functions. The mild nutrient stress it creates first optimizes the plant-AMF symbiosis, which in turn reshapes the rhizosphere microenvironment. This altered environment selectively guides the assembly of a complex and cooperative microbial network, leading to the formation of a functionally stratified microbiome orchestrated by keystone taxa (Banerjee et al., 2018). Although this mechanism has been validated under specific field conditions in this study, its long-term stability and broader applicability require further confirmation through multi-location trials. Future research should employ manipulative experiments, such as synthetic microbial community inoculation, to clarify causal relationships and explore the potential for integrating this strategy with other sustainable agronomic practices.

## 5 Conclusions

In this study, we demonstrate that the integration of a moderate fertilizer reduction (85% of conventional input) with ECF triggers a multi-dimensional functional reprogramming of the wheat rhizosphere microbiome, spanning community structure, network architecture, and metabolic activity. The treatment promoted a nutrient dependent microbial assembly, which enhanced the complexity of fungal ecological networks while simplifying bacterial network interactions. Notably, it activated microbial carbon and nitrogen cycling potential and enriched keystone taxa, including *Trichoderma*, *Solicoccozyma*, and *Candidatus_Solibacter*, which were strongly correlated with soil ecosystem functions. These coordinated shifts collectively enhanced soil nutrient cycling functions and supported plant growth under reduced fertilization. Our findings offer a reliable microbiome-based strategy for achieving sustainable wheat production while preserving soil fertility, providing novel insights for the development of targeted microbial management practices in agroecosystems.

## Data Availability

The data presented in the study are deposited in the NCBI Sequence Read Archive (SRA) repository, under project accession numbers PRJNA1412241 and PRJNA1412239.
