# Supplementary material for: Ecological composite fertilizer application enhances wheat yield and optimizes rhizosphere microbial community under reduced fertilization
Source: Front Plant Sci. 2026 Feb 2;17:1771450. doi: 10.3389/fpls.2026.1771450 (PMC12907401; doi:10.3389/fpls.2026.1771450)
Supplement: Supplementary file 1 [file DataSheet1.docx]

**Supplementary Material**

**Ecological composite fertilizer application enhances wheat yield and rhizosphere microbial community under reduced fertilization**

**Yu Wang^1,2^, Xinhao Luo^1,2^,** **Meiling Ping^1,2^, Haining Wang^1,2^, Yueming Bao^1,2^, Chuansheng Zhao^1,2^, Xiaoyu Li^1,2, *^, Jin Chen^1,2, *^**

^1^ Schools of Life Sciences, Anhui Agricultural University, Hefei, 230036, China.

^2^ National Engineering Laboratory of Crop Stress Resistance Breeding, Anhui Agricultural University, Hefei, 230036, China.

*** Correspondence:**Corresponding Author
Jin Chen, Schools of Life Sciences, Anhui Agricultural University, Hefei, Anhui, 230036, China. E-mail address: chenjin131721@ahau.edu.cn

Xiaoyu Li, Schools of Life Sciences, Anhui Agricultural University, Hefei, Anhui, 230036, China. E-mail address: lixiaoyu@ahau.edu.cn

Supporting Information Includes:

- Number of pages: 5
- Number of Tables: 1
- Number of Figures: 2

**Text 1** Quantitative PCR (qPCR) analysis.

We quantified the copy numbers of bacterial 16S rRNA genes, fungal internal transcribed spacer (ITS) regions, and key functional genes involved in carbon (*cbbLR*), nitrogen (*amoA*), and phosphorus (*phoD*) cycling across different soil treatment groups using quantitative PCR (qPCR). The qPCR assays were performed on a Bio-Rad CFX Connect Real-Time System (Bio-Rad, USA) using SYBR Green chemistry. Specific primer pairs for each target gene (see Table S1) were used. Each 10 μL reaction contained 5 μL of 2× SYBR Green qPCR Master Mix, 0.5 μL each of forward and reverse primers (10 μM), 1 μL of template DNA, and 3 μL of double-distilled H₂O. Reactions were assembled in a 96-well PCR plate (AG12101, Accurate Biotechnology, China). The thermal cycling program consisted of an initial denaturation at 95 °C for 7 min, followed by 40 cycles of 95 °C for 5 s and 60 °C for 30 s, concluding with a melt curve analysis. Diethylpyrocarbonate (DEPC)-treated water served as the no-template control, and positive DNA controls were included in each run. Standard curves were generated using serial dilutions of plasmids containing the target gene fragments, with R² values > 0.99. All reactions were performed in quadruplicate, and amplification efficiencies ranged from 80.1% to 90.9%.

**Table S1** List of the primers used for quantitative PCR.

| **Gene** | **Forward sequence (5’-3’)** | **Reverse sequence (5’-3’)** | **Size (bp)** |
| --- | --- | --- | --- |
| 16S rRNA | 338F(ACTCCTACGGGAGGCAGCAG) | 518R(ATTACCGCGGCTGCTGG) | 200 |
| ITS | ITS3F(GCATCGATGAAGAACGCAGC) | ITS4R(TCCTCCGCTTATTGATATGC) | 340 |
| *cbbLR* | AAGGAYGACGAGAACATC | TCGGTCGGSGTGTAGTTGAA | 815 |
| *amoA* | GGGGTTTCTACTGGTGGT | CCCCTCKGSAAAGCCTTCTTC | 491 |
| *phoD* | CAGTGGGACGACCACGAGGT | GAGGCCGATCGGCATGTCG | 371 |

**
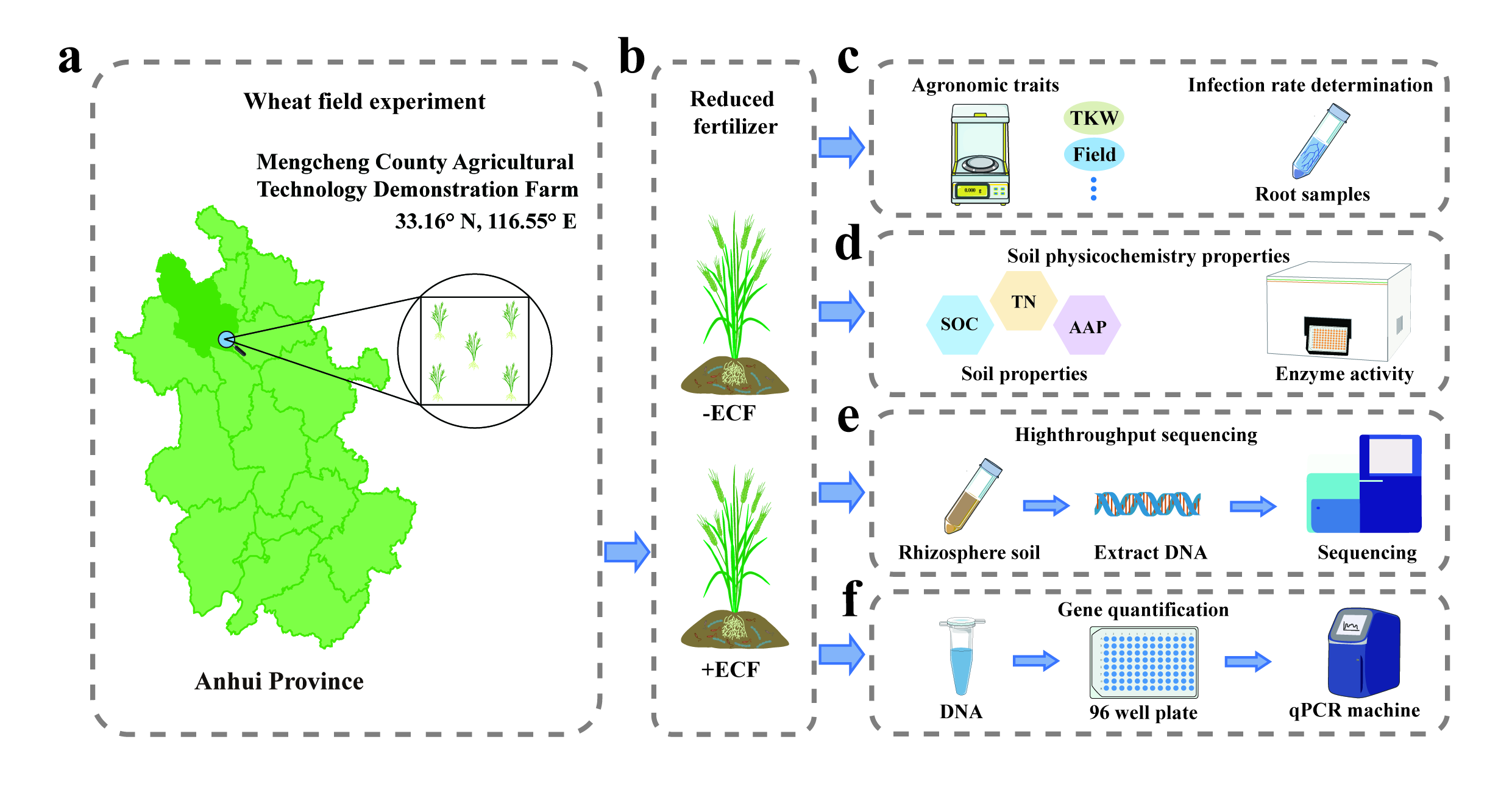
 Figure S1 Flowchart of wheat field trials and subsequent procedures.** Samples of wheat plants and rhizosphere soil from different treatment groups were collected for subsequent experiments (a, b). Wheat plant samples were used for phenotypic data measurement and root colonization rates determination (c). Rhizosphere soil was primarily preserved in three parts: the first part was stored at room temperature to dry naturally, used for determining soil physicochemical properties (d); the second part was stored at 4°C, used for measuring spore density; the third part was stored at -80°C, used for extracting genomic DNA, and subsequently subjected to microbial high throughput sequencing (e) and qPCR analysis (f).

**
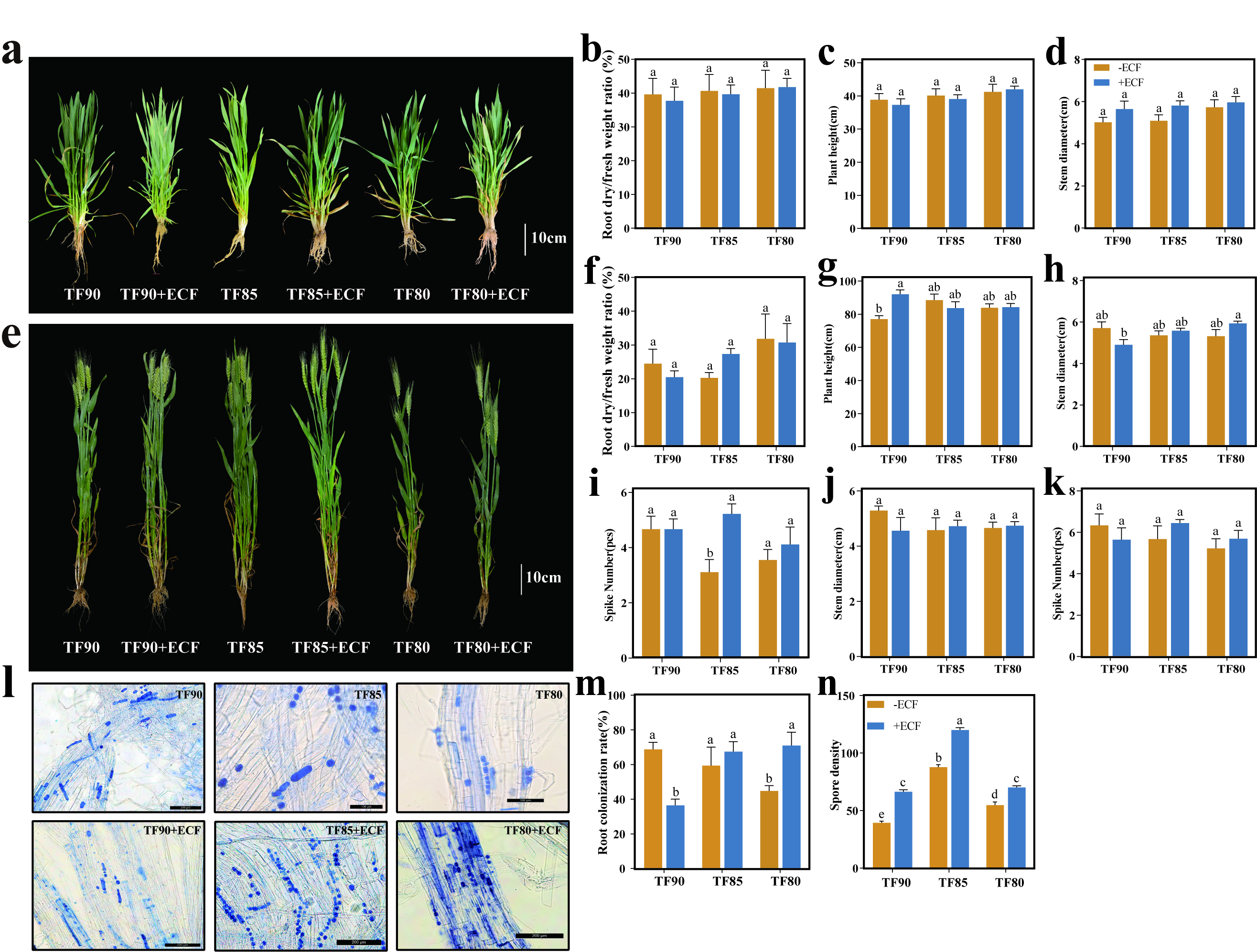
**

**Figure S2** **Effects of fertilizer reduction combined with ECF on wheat agronomic traits and root colonization rate.** (a,e) Phenotypic images of wheat plants at the jointing and flowering stages. (b-d) root dry/fresh weight ratio, plant height and stem diameter at jointing stage. (f-i) root dry/fresh weight ratio, plant height, stem diameter and spike number at flowering stage. (j, k) stem diameter and spike number at maturity period. (l-n) Root colonization rate and spore density. Data represent mean ± SD (n≥3). Different superscript letters denote statistically significant differences among treatments (*P < 0.05*).
